# The Quality Characteristics of Gluten-Free Cake Produced from Maize Starch, Chia and Lentil Flours

**DOI:** 10.1007/s11130-025-01354-3

**Published:** 2025-05-02

**Authors:** Aynur Kılıç, Hüseyin Boz

**Affiliations:** Gastronomy and Culinary Arts Department, Tourism Faculty, Erzurum, 25240 Turkey

**Keywords:** Physical properties, Textural properties, Sensorial properties, Storage

## Abstract

The aim of this study was to analyze the effect of chia (0–4 and 8%) and lentil flour (0–8 and 16%) on the physical, sensorial, and textural properties of maize starch cake. The results showed that the addition of gluten-free ingredients decreased the internal color L* and a* color values ​​of cakes but increased the b* color values. In general, gluten-free ingredients positively affected the symmetry properties of the cake samples and symmetry index values of gluten-free cakes varied between 8.5 and 24.5 mm. The addition of chia flour decreased the hardness of the cake samples on the first day. Gluten-free ingredients caused a decrease in the hardness of the cake samples on all three days of storage. Adding chia and lentil flour to gluten-free cake formulations did not affect the chewiness of the cakes on the first day of storage, but reduced the chewiness of gluten-free cakes on the second and third days of storage. In the sensory evaluation, the most appreciated formulation by the panelists was the formulation containing 4% chia and 8% lentil flour. The results show that the properties of maize starch cake can be improved by the addition of chia and lentil flours.

## Introduction

In recent years, growing awareness of celiac disease, scientific research on gluten-free products has increased [1–3]. This disease, which damages the small intestines and can affect the normal absorption of other nutrient components, is gluten intolerance, which can develop in people with a genetic predisposition who consume foods containing gluten [2]. It is stated that the incidence of celiac disease is approximately 1.4% in the world, and the only cure for this disease is to maintain a strict gluten-free diet [3–5].

Chia (*Salvia hispanica* L.) is a plant that produces oil seeds from the *Lamiaceae* family, widely cultivated in Latin America and Australia. Its seeds are a product that has been available on the European market since 2009 and are permitted to be commercialized as “Novel Food” for various uses listed in EU Commission Implementing Regulation (EU) 2017/2470 [6–8]. Chia exhibits approximately 486 kcal/100 g, 30% total lipids, 16% protein, 34% dietary fiber and 42% carbohydrates. It is also an important source of high concentrations of important nutrients such as phenolic compounds, vitamins, minerals, and unsaturated fatty acids [9, 10]. Previous studies show that chia seeds are widely used in the production of bakery products such as biscuits and bread [11, 12].

Legumes are known as an important food source because they contain high amounts of protein, carbohydrates and fiber. In addition, these products contain significant levels of vitamins, minerals and essential amino acids and contain two to three times more protein (18–31.6%) than grains. For this reason, legumes, which have an important place in human nutrition, have been used in many studies to enrich the nutritional content of bakery products such as bread [13, 14].

The most important challenge in producing gluten-free products is to formulate them with the same nutritional value and textural properties as gluten-containing foods. Today, many different products can be produced for people with gluten sensitivity, and these products are easily accessible as they are sold in markets almost everywhere. However, the absence of gluten in the flours used in the preparation of foods required for celiac patients creates deficiencies in sensory and textural properties of these products and negatively affects the product quality. These negative effects in gluten-free products are tried to be eliminated by using various additives. These substances used in the preparation of foods required for celiac patients can cause both people with gluten sensitivity and their parents to have negative thoughts about their health. Using only starch in the preparation of gluten-free cakes causes these products to be deficient in terms of nutritional content. In addition, cakes containing only starch should be consumed on the same day as they become stale early due to the retrogradation of starch. It was hypothesized that when chia and bean flour, which contain both dietary fiber and protein, are added to maize starch cake formulations, starch retrogradation can be slowed down and the cake samples can be given sensory and textural properties that can be easily consumed in the following days. By adding chia seeds and green lentil flour to gluten-free maize starch, the nutritional content of gluten-free cake can be enriched and an alternative product can be produced. This study aimed to determine the effect of partially replacing maize starch with chia seeds and green lentil flour on the physical, textural and sensorial properties of starch cakes.

## Materials and Methods

### Materials

Maize starch (protein 0.4%, moisture content 10.8%, and lipid 0.6%, Dola gluten free starch, Türkiye), defatted chia seed flour (dietary fiber 29.62 mg/100 g, carbohydrate 37.7%, and moisture content 5.8%, Tazemiz chia seed flour, Turkey), and green lentil flour (carbohydrate 36.62%, protein 23%, lipid content 0.92%, and dietary fiber 25.99 mg/100 g, İpek, Türkiye) were purchased from local and national markets. Other ingredients used in cake production, such as oil, eggs, sugar, baking powder and salt, were also supplied from the market in Erzurum, Türkiye.

### Gluten-Free Cake Making Procedure

In the production of maize starch cake, 120 g maize starch/chia flour/lentil flour, 92 g sugar, 92 g oil, 120 g whole eggs, 45 mL milk, 0.5 g salt and 1.5 g baking powder were used. The cake formulations were prepared based on maize starch amount. Only maize starch was used in the production of control gluten-free cake, and the amounts of chia (0–4 and 8%) and lentil flour (0–8 and 16%) added to the cake formulations were deducted from the amount of maize starch.

During the preparation of the cake dough, the mixing of the dough components was carried out in a certain order. First, whole eggs and salt were mixed at speed 1 of the mixer (Tefal, Turkey) for about 3 min. After adding sugar, it was mixed for 1 min. Milk and oil were added and mixed under the same conditions for 1 min. All dry ingredients used in making the cake (maize starch/chia seed flour/green lentil flour and baking powder) were added together and mixed at the same speed for 3 min until the cake dough was obtained. After the resulting cake dough was placed in metal cake molds (30 g), it was baked in an electric oven at 195 °C for 23 min without turning on the oven fan. Following the baking process, the cake samples were cooled to room temperature (22–24 °C) for approximately 1 h. The cake samples were then covered with plastic stretch film and stored at room temperature (22–24 °C) for three days.

### Determination of Physical Characteristics

Crust color and crumb color measurements of the cake samples were determined with a Minolta (CR-200, Japan) colorimeter. The triple scale consisting of L*, a*, and b* values was taken into account as color values. L = 100 lightness, L = 0 darkness; (+) a red, (-) a green; (+) b was evaluated as yellow and (-) b as blue [15]. Specific volume values of cakes were calculated by dividing the cake volume by the weight of the cake. Volume measurement in cake samples cooled at room temperature for one hour was determined by dipping the cakes covered with stretch film into a container filled with water and measuring the amount of overflowing water as volume. Specific volume values in the cake samples were determined as ml/gram according to the weight and volume results obtained from the cake samples. Volume index and uniformity index values of the cake samples were determined by using the American Association of Cereal Chemists (AACC 10–91) procedure [16].

### Determination of Textural Properties

Texture analysis on gluten-free cake samples were made with the TA-XT Plus texture analyzer (TA-XT plus; Stable Micro System Ltd., Godalming, UK) with a 50 mm probe [17]. Texture analysis was performed on cylindrical samples with a height of 1.5 cm and a diameter of 2.3 cm, taken from the middle point of the cakes. Cylindrical samples were taken from the center of the cake crumb. The test conditions for texture analysis were as follows: 1 mm/s pre-test, test and post-test speed, strain %40. The results obtained as a result of texture analysis were expressed as hardness, springiness, cohesiveness and chewiness. Texture profile analyzes of the gluten-free cake samples on the first day were carried out two hours after the baking process, and care was taken to complete the 24-h period for the second and third day analyses.

### Sensory Evaluation

After the produced cake samples were cooled, they were sensory evaluated by 20 panelists consisting of academic staff working in the Department of Gastronomy and Culinary Arts, and master’s and doctoral students. Panelists were asked to rate cake samples on sensory parameters (taste, aroma, color, texture and overall acceptability) using a 9-point hedonic scale. (1 = dislike extremely to 9 = like extremely). The cake samples to be evaluated sensory were randomly coded with three-digit numbers on plastic plates and presented to the panelists accompanied by drinking water [18].

### Statistical Analysis

The results obtained from the study were analyzed as two-way analysis of variance (ANOVA). Significant means (*p* < 0.05) of the main sources of variation were compared with the Duncan Multiple Comparison Test (version 22.0; SPSS Inc., Chicago, IL, USA). Two-way ANOVA was used to determine interactions between the tested variables. Data obtained as a result of statistical analysis are expressed as mean ± standard error.

## Results and Discussion

### Physically Properties of Cakes

In general, the color of bakery products may vary depending on the physicochemical properties of the dough, baking time and temperature. Maillard and caramelization reactions that occur depending on these parameters are the most important reactions that are effective in the formation of color in bakery products [19]. As shown in Table 1, the addition of chia flour affected the crust color L* values of the cake samples at a level of *p* < 0.05. While chia flour did not cause a significant change in the crust color L* values of the cakes, the addition of lentil flour at 8% chia flour level caused a decrease in the L* color values of the cakes. A significant decrease in the a* color values of the cake samples was detected with the addition of chia flour. The highest a* color value was determined in the formulations containing 0% chia flour and 16% lentil flour, and the lowest a* color values were determined in the cake samples containing 8% chia flour and 0% lentil flour. Similar results were obtained in cake samples produced using wheat flour, and a decrease in the color values of cake samples was determined with the addition of 7% chia flour [20]. On the other hand, a decrease in the b* color values of the crust color of the cake samples was observed with the addition of chia flour, and an increase was observed with the addition of lentil flour. Results similar to our study were obtained in a study in which gluten-free cake was produced by using chia and quinoa flours together, and it was determined that the addition of chia flour caused a decrease in the b* color values of the cakes [21]. The rich composition of chia flour may have accelerated the Maillard reaction in terms of crust color formation and increased the darkness of the crust color.

**Table 1:** The effect of chia and lentil flours on the color parameters of maize starch cake

CF (%)	LF (%)	Crust color			Crumb Color		
		L*	a*	b*	L*	a*	b*
0	0	47.26 ± 0.68bc	18.43 ± 0.06c	16.30 ± 0.31 cd	76.03 ± 0.79a	0.08 ± 0.03d	18.80 ± 0.02b
	8	45.06 ± 0.17c	18.84 ± 0.05b	13.93 ± 0.37e	69.73 ± 0.25b	1.03 ± 0.01c	18.48 ± 0.57b
	16	50.68 ± 0.63a	20.50 ± 0.07a	22.19 ± 0.05a	65.71 ± 0.44c	0.93 ± 0.08c	20.23 ± 0.20a
4	0	48.45 ± 0.47b	17.05 ± 0.08e	19.05 ± 1.00b	64.30 ± 0.20d	3.56 ± 0.25b	12.46 ± d0.16e
	8	47.51 ± 0.30bc	17.19 ± 0.15de	13.47 ± 0.07e	61.72 ± 0.03e	3.40 ± 0.05b	12.75 ± 0.54 cd
	16	46.63 ± 0.05c	17.44 ± 0.05d	12.60 ± 0.20e	60.31 ± 0.55f	3.56 ± 0.11b	13.42 ± 0.02c
8	0	47.61 ± 0.36bc	16.47 ± 0.05f	17.30 ± 0.09c	59.73 ± f0.13 g	4.37 ± 0.05a	10.38 ± 0.05 g
	8	48.03 ± 0.62bc	16.96 ± 0.01e	17.45 ± 0.39c	59.30 ± 0.30 fg	4.38 ± 0.09a	11.33 ± 0.05f
	16	47.05 ± 0.35bc	16.63 ± 0.17f	15.56 ± 0.42d	58.41 ± 0.33 g	4.21 ± 0.05a	11.57 ± 0.12ef
		*	**	**	**	**	**

Values are mean ± SE. Different letters in the same column are significantly different (*P < 0.05; **P < 0.01), CF; chia flour, LF; lentil flour

The addition of lentil flour to gluten-free cake formulations caused a decrease in the crumb color L* values of the cake samples. This decrease in the crumb color L* values ​​of the cake samples may be due to the chia and lentil flours used as raw materials being darker in color than maize starch. In a study, results similar to those obtained in our study were obtained and it was determined that the addition of lentil flour darkened the crumb color of cake samples [22]. The crumb color a* values of the cakes increased with the addition of lentil flour, and the highest a* color values were determined in the cake samples containing 8% lentil flour. The addition of 16% lentil flour did not have a different effect on a* color values compared to the 8% level. The b* color values of the cakes increased with the addition of lentil flour, and the highest crumb color b* values were reached in the formulations containing 16% lentil flour.

Volume is an important quality criterion that significantly affects consumer preference for cakes and is directly related to the type and quantity of ingredients used in the production of the cake [23]. Cake samples that are as voluminous, symmetrical and uniform as possible are considered to be of high quality, and it is stated that quality cakes should be slightly circular (round), as symmetrical as possible, have high volume and low shrinkage values. The addition of chia flour caused an increase in the specific volume values of gluten-free cake samples at the 4% level, but did not cause a significant change in the specific volume values at the 8% chia flour level compared to the 4% chia flour level (Table 2). Among the cake samples, the lowest specific volume values were reached in the control cake samples that did not contain chia flour.

**Table 2:** The effect of chia and lentil flours on the physical properties of maize starch cake

CF (%)	LF (%)	Specific volume(g/ml)	Volume index(mm)	Uniformity index(mm)	Symmetry index(mm)
0	0	2.91 ± 0.04c	94.0 ± 1.00cd	1.0 ± 1.50a	11.5 ± 1.50cd
	8	3.06 ± 0.10abc	91.5 ± 1.50d	1.5 ± 0.50a	14.0 ± 1.00c
	16	3.05 ± 0.04abc	93.5 ± 2.50cd	0.5 ± 0.50a	8.5 ± 0.50e
4	0	3.02 ± 0.07bc	98.5 ± 0.50ab	0.5 ± 0.50a	9.5 ± 0.50de
	8	3.34 ± 0.13a	95.0 ± 1.00bcd	0.5 ± 0.50a	9.5 ± 0.50de
	16	3.26 ± 0.11ab	97.5 ± 00.50abc	1.0 ± 0.00a	19.0 ± 1.00b
8	0	3.10 ± 0.08abc	99.5 ± 0.50a	1.5 ± 0.50a	24.5 ± 0.50a
	8	3.15 ± 0.04abc	101.0 ± 1.00a	0.5 ± 0.50a	21.5 ± 0.50b
	16	3.33 ± 0.11a	99.5 ± 0.50a	1.0 ± 0.00a	20.5 ± 0.50b
		ns	**	ns	**

Values are mean ± SE. Different letters in the same column are significantly different (*P < 0.05; **P < 0.01), CF; chia flour, LF; lentil flour

It is desirable that the uniformity index, which is based on measuring the difference between the heights of the two end points of cake samples, be as close to zero as possible. Because this indicates that the dough grows uniformly and the cake is structurally protected during baking and cooling. In our study, it was determined that the addition of chia and lentil flours did not cause a significant change in the uniformity index values of the cake samples.

The symmetrical development of the dough during baking is an important feature in cakes, and therefore the formation of a peak in the middle of the cake is desirable due to the rise that occurs during baking. Therefore, the symmetry index values of cakes should be greater than zero, indicating that the center is higher than the extremes [23]. Symmetry index values of gluten-free cake samples vary between 8.5 and 24.5 mm (Table 2). Positive symmetry index values indicate that there is no inward collapse in the cake samples. Therefore, it can be said that the gluten-free ingredients added to the cake formulations positively affect the symmetry properties of the maize starch cake.

### Textural Properties of Cakes

Hardness is considered one of the most notable textural parameters in the evaluation of bakery products, as it is closely related to consumers’ perception of freshness [24]. It is seen that the addition of chia flour caused a decrease in the hardness of the cake samples on the first day (Table 3). This may be due to the fact that chia flour reduces water loss in cake samples due to its high dietary fiber content. On the second day of storage, it was observed that the addition of 4% chia flour decreased the hardness slightly compared to the control cake samples, but the addition of 8% chia flour decreased the hardness as on the first day. It was determined that the addition of 10% chia flour in cake samples produced using rice flour caused a decrease in the hardness of the cake samples [25]. In another study, similar results were obtained and it was determined that the addition of chia flour in breads produced from wheat flour caused a decrease in the hardness of the breads [26]. On the other hand, considering three different lentil flour levels, the highest hardness was determined in the control samples that did not contain lentil flour, and the lowest hardness was determined in the cake samples containing 16% lentil flour. The addition of lentil flour on all three days of storage generally resulted in a decrease in the hardness of the cake samples. In other words, it can be said that lentil flour reduces water loss in cake samples produced from maize starch. Gülhan and Karaça [27] obtained similar results in their study and observed that the hardness in cake samples decreased with the addition of lentil flour to cake formulations.

**Table 3:** The effect of chia and lentil flours on hardness and springiness of maize starch cake

CF (%)	LF (%)	Hardness (N)			Springiness		
		1st day	2nd day	3rd day	1st day	2nd day	3rd day
0	0	3.18 ± 0.05a	8.47 ± 0.05a	10.19 ± 0.19a	0.002 ± 0.001b	0.064 ± 0.004a	0.108 ± 0.004a
	8	2.54 ± 0.01c	4.95 ± 0.03g	7.79 ± 0.11d	0.001 ± 0.000b	0.028 ± 0.003b	0.033 ± 0.005cd
	16	2.86 ± 0.12b	6.00 ± 0.11e	9.56 ± 0.05b	0.002 ± 0.001b	0.056 ± 0.004a	0.099 ± 0.002a
4	0	3.06 ± 0.05a	7.28 ± 0.05c	9.75 ± 0.11ab	0.002 ± 0.001b	0.016 ± 0.002cd	0.066 ± 0.006
	8	2.67 ± 0.02c	7.66 ± 0.05b	9.54 ± 0.15b	0.003 ± 0.001ab	0.028 ± 0.002b	0.069 ± 0.008b
	16	1.84 ± 0.03d	5.32 ± 0.16f	8.50 ± 0.07c	0.001 ± 0.000b	0.010 ± 0.002d	0.066 ± 0.002b
8	0	1.92 ± 0.02d	6.36 ± 0.01d	7.33 ± 0.32de	0.003 ± 0.001ab	0.021 ± 0.001bc	0.044 ± 0.003c
	8	1.86 ± 0.01d	4.54 ± 0.03h	7.44 ± 0.29de	0.004 ± 0.001a	0.014 ± 0.002cd	0.029 ± 0.003d
	16	1.52 ± 0.01e	4.43 ± 0.06h	6.97 ± 0.01f	0.004 ± 0.001a	0.009 ± 0.001d	0.029 ± 0.002d
		**	**	**	ns	**	**

Values are mean ± SE. Different letters in the same column are significantly different (*P < 0.05; **P < 0.01), CF; chia flour, LF; lentil flour

In general, the springiness of the cake samples increased with storage time. The addition of chia and lentil flours to the cake formulations on the first day of storage increased the springiness of the cake samples to a limited extent, but this increase was not at a statistically significant level (Table 3). On the second day of storage, both chia flour and lentil flour generally caused a decrease in the springiness of the cake samples. Considering that high springiness is desired, it can be said that the addition of chia and lentil flour has a negative effect. In a study, similar results were obtained and it was determined that the addition of lentil flour caused a decrease in the elasticity of sponge cake samples [22]. In addition, on the third day of storage, the variables generally caused a decrease in the elasticity of the cake samples (except when using 4% chia and 8% lentil flour).

Cohesiveness defined as a concept that expresses the density of internal bonds in the structure of food [28]. In general, the cohesiveness of the cake samples decreased with storage time. On the first day of storage, the addition of lentil flour at the level of 4% chia flour increased the cohesiveness of the cake samples (Table 4). Additionally, the addition of 16% lentil flour at the 8% chia flour level caused an increase in the cohesiveness of the cake samples. On the second day of storage, the highest cohesiveness was determined in formulations containing 4% and 8% chia flour. On the other hand, it was determined that the addition of chia flour in gluten-free cakes produced from rice flour caused a decrease in cohesiveness in cake samples, contrary to the results obtained in our study [25].

**Table 4:** The effect of Chia and lentil flours on cohesiveness and chewiness values of maize starch cake

CF (%)	LF (%)	Cohesiveness			Chewiness(N)		
		1st day	2nd day	3rd day	1st day	2nd day	3rd day
0	0	0.77 ± 0.03bc	0.48 ± 0.02de	0.43 ± 0.01cd	0.003 ± 0.001a	0.240 ± 0.02a	0.425 ± 0.01a
	8	0.78 ± 0.02ab	0.51 ± 0.02cd	0.54 ± 0.04a	0.002 ± 0.001a	0.090 ± 0.01bcd	0.345 ± 0.01b
	16	0.74 ± 0.02cd	0.44 ± 0.02e	0.41 ± 0.02d	0.002 ± 0.001a	0.120 ± 0.01b	0.370 ± 0.01b
4	0	0.71 ± 0.00d	0.52 ± 0.01c	0.41 ± 0.02d	0.002 ± 0.001a	0.065 ± 0.01de	0.235 ± 0.01d
	8	0.76 ± 0.01bc	0.54 ± 0.01bc	0.46 ± 0.01bc	0.002 ± 0.001a	0.110 ± 0.01c	0.315 ± 0.02c
	16	0.82 ± 0.01a	0.59 ± 0.01a	0.43 ± 0.01cd	0.001 ± 0.000a	0.045 ± 0.01e	0.225 ± 0.02d
8	0	0.81 ± 0.01a	0.59 ± 0.01a	0.50 ± 0.01ab	0.002 ± 0.001a	0.075 ± 0.01de	0.165 ± 0.01e
	8	0.78 ± 0.01ab	0.57 ± 0.01ab	0.50 ± 0.01ab	0.002 ± 0.001a	0.095 ± 0.01abc	0.120 ± 0.01f
	16	0.82 ± 0.01a	0.58 ± 0.01ab	0.51 ± 0.01a	0.002 ± 0.001a	0.085 ± 0.01cd	0.115 ± 0.01f
		**	**	**	ns	**	**

Values are mean ± SE. Different letters in the same column are significantly different (*P < 0.05; **P < 0.01), CF; chia flour, LF; lentil flour

Chewiness can be defined as the energy required to physically break a solid food into pieces and make it ready for swallowing, or as the number of chewing times required until the food is ready for swallowing [29]. The chewiness of the cake samples increased depending on storage, and the highest chewiness was detected after three days of storage. On the first day of storage, the addition of chia and lentil flour to the gluten-free cake formulations did not affect the chewiness of the cake samples, but they reduced the chewiness of the gluten-free cake samples on the second and third days of storage (Table 4). In light of these results, it can be stated that the addition of chia and lentil flour reduces the energy required to make the cake samples ready to swallow. In a study, it was reported that the chewiness increased with the addition of chickpea, pea and bean flours to the rice flour-based cake formulation, while the addition of lentil flour did not cause any change in chewiness [30].

### Sensory Properties of Cakes

**Fig. 1:**
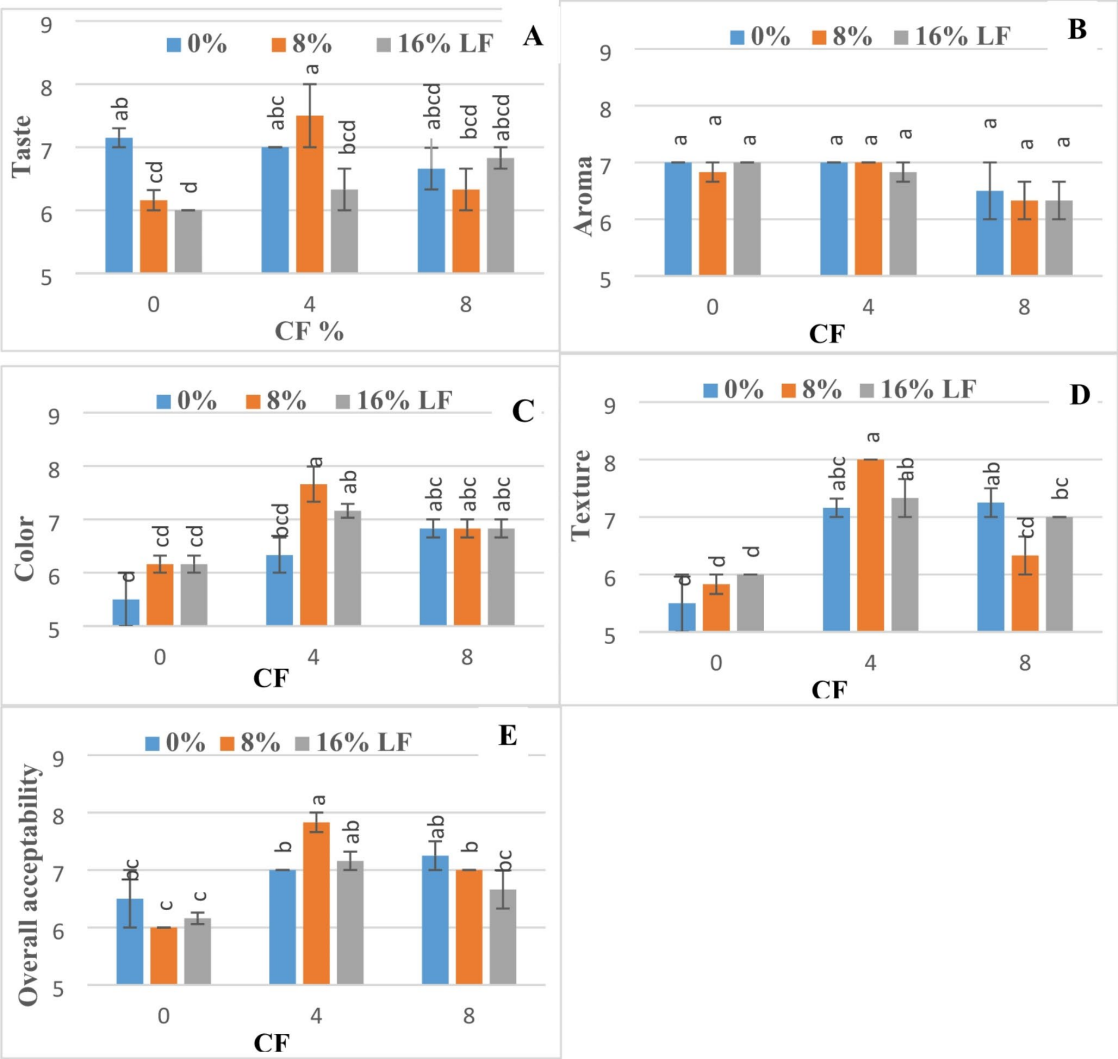
The effect of chia and lentil flours on the sensorial parameters of maize starch cake. Different letters on the bars are significantly different (*P* < 0.05). CF: Chia seed flour, LF; Lentil flour

Chia and lentil flours usage decreased the taste score of gluten-free cakes (except when using 4% chia and 8% lentil flour) (Fig. 1). The cakes with 4% chia flour and 18% lentil flour received the highest taste score in all cake samples. On the other hand, the variables did not create a statistically significant difference in the aroma values of the cake samples. The rich composition of chia and lentil flours compared to maize starch positively affected the color values of the cake samples. All of the cake samples containing chia and lentil flour achieved higher sensory color scores compared to the control cake sample containing 100% maize starch. All of the formulations were accepted by the panelists in terms of texture, but the highest texture scores were achieved in the cake samples containing 4% chia flour and 8% lentil flour. Moreover, the cake samples containing chia and lentil flours all achieved higher overall acceptability scores compared to the control cake sample containing 100% maize starch. According to results of sensorial evaluation, the formulations containing 4% chia and 8% lentil flours are identified as the best cake formulations.

It is clear that the addition of chia and lentil flour to cake formulations produced from maize starch will enrich the nutritional content of the cake samples. According to the results obtained from this study, it can be said that the addition of chia and lentil flours improves the physical, textural and sensory properties of maize starch cake. The cake samples produced with the addition of gluten-free cha and lentil flours gained sensory and textural properties that could be easily consumed not only on the first day but also on the second and third days.

## Conclusions

This study showed that the addition of chia and lentil flour substantially improved the physical, textural and sensory properties of maize starch cake. By incorporating chia and lentil flour into maize starch cake formulations, it is possible to obtain a product with good textural and sensory performance. It is possible to incorporate chia and lentil flour into maize starch cake formulations and obtain a product with good technological and sensory performances. The presence of chia and lentil flour generally positively affects the specific volume, symmetry properties, color parameters and hardness of maize starch cakes. According to the sensory evaluation results, formulations containing 4% chia and 8% lentil flour were determined to be the most appreciated by the panelists.

## Data Availability

No datasets were generated or analysed during the current study.
